# Technical Note: Novel Use of CytoSorb™ Haemadsorption to Provide Wound Healing Support in Case of Severe Burn Trauma *via* Reduction of Hyperbilirubinaemia

**DOI:** 10.3389/fsurg.2021.743571

**Published:** 2021-12-17

**Authors:** Katarzyna Rachunek, Maja Krause, Johannes Tobias Thiel, Jonas Kolbenschlag, Adrien Daigeler, Andreas Bury

**Affiliations:** ^1^Department of Hand, Plastic, Reconstructive and Burn Surgery, BG Trauma Center, Eberhard Karls University of Tuebingen, Tuebingen, Germany; ^2^Department of Anesthesiology and Intensive Care Medicine, BG Trauma Center, Eberhard Karls University of Tuebingen, Tuebingen, Germany

**Keywords:** secondary sclerosing cholangitis, liver failure, burn trauma, CytoSorb, hemoadsorption

## Abstract

Hyperbilirubinaemia has been shown to compromise wound healing in severely burned patients. The therapy options for patients with impairment of wound healing and subsequent severe liver dysfunction are limited. A novel extracorporeal treatment, CytoSorb^®^ (CytoSorbents Corp, USA), is a whole blood adsorber composed of highly biocompatible and porous polystyrene divinylbenzene copolymer beads covered in a polyvinylpyrrolidone coating. It is capable of extracting mainly hydrophobic middle-sized (up to 55 kDa) molecules from blood *via* size exclusion, including cytokines and bilirubin. We performed therapy with CytoSorb^®^ on a severely burned (48% Total Body Surface Area-TBSA) patient with secondary sclerosing cholangitis (SCC) to promote the wound healing process by reducing bilirubin concentrations and to bridge the time to spontaneous liver regeneration or eventually to liver transplantation after two skin transplantations had failed to provide wound closure. In the first 6 days the cartridge was changed on a daily basis and later after every 2–4 days. The therapy with six adsorbers decreased a total bilirubin concentration from 14.02 to 4.29 mg/dl. By maintaining a stable bilirubin concentration under 5 mg/dl, debridement of abdomen and upper extremities with autologous skin grafting and, 4 weeks later, autologous skin grafting of the back from scrotum and lower extremities were performed successfully. After wound healing had been achieved, the CytoSorb therapy was discontinued after 57 days and 27 adsorber changes. CytoSorb therapy can be a promising support of wound and skin graft healing in patients with severe burns and liver dysfunction due to a significant reduction of total bilirubin concentration.

## Background

An obstructive jaundice can have a negative effect on wound healing due to delay in fibroblast proliferation, collagen deposition and immunological response (1–3). The unconjugated bilirubin itself was shown to suppress the growth of fibroblast colonies in *in vitro* studies (4, 5). Moreover, wound complications in patients with jaundice, in patients with severe burns and hyperbilirubinaemia and in cases of abdominal surgery were shown to be more common than in patients without liver dysfunction (6–8).

Patients suffering severe burns are at risk of developing cholangiopathies, as burn associated cholestasis (BAC) occurs in about half of them (9). BAC doubles the risk of mortality during the intensive care unit (ICU) stay and is associated with bacteraemia and extrahepatic organ failure (9–11). It can progress to secondary sclerosing cholangitis (SSC), which represents a progressive and extremely destructive cholestatic biliary disorder with poor prognosis (12). Due to rising awareness, this disease has been increasingly observed in patients with long treatment stays in ICU; and it is called secondary sclerosing cholangitis in critically ill patients (SSC-CIP) (13). Mechanisms leading to cholangiopathy in critically ill patients, including severely burned patients, remain poorly understood; however, current data suggest that the triggering events are persistent biliary obstruction, ischemic injury and surgical trauma to the bile duct or biliary tree. The disease is characterized by inflammation, obliterative fibrosis with progressive destruction of the intrahepatic biliary tree, and biliary casts and stricture formation, which ultimately leads to biliary cirrhosis and liver failure (14, 15).

A burn trauma in combination with cholangiopathies trigger a vicious cycle with liver dysfunction delaying wound healing, and wounds promoting sepsis, hypotonia and further liver injury. Despite the fact that the liver, unlike any other organ, has an enormous regenerative potential, it cannot restore its function under such critical circumstances. The therapeutic strategies for such patients are limited.

Additionally, patients with severe burn injuries are at immediate risk of burn shock and a systemic hyperinflammatory reaction due to an overwhelming immune and inflammatory response particularly in the early phase after the accident (16). The local burn injury leads to capillary damage with systemic release of vasoactive substances, such as prostaglandins, histamines, cytokines, with a consecutive increase in permeability for blood proteins, resulting in capillary leakage. This results in a generalized edema with a concomitant massive intravascular loss of water, electrolytes and proteins which can be further exacerbated by wound exudation. The resulting loss of intravascular volume (hypovolaemia) in turn leads to perfusion disturbances in the peripheral microcirculation leading to metabolic acidosis, hemodynamic instability and ultimately organ dysfunction and death. The course of the disease is dynamic and requires immediate intensive care and surgical measures to stabilize the patient, ensure survival, prevent secondary damage and create the best possible conditions for rehabilitation (17). Other risks for patients with severe burns are arising from burn wound infections and a high percentage of deaths in these patients seems to be related to sepsis or other infection complications (18).

Several publications have reported that haemoadsorption therapy using the Cytosorb device resulted in a reduction of inflammatory parameters (C-reactive protein [CRP], procalcitonin [PCT], Interleukin [IL]-6, tumor necrosis factor [TNF]-α) as well as in hemodynamic stabilization with a concomitant reduction in vasopressor requirements in septic patients but also in those with non-infectious origins (19–21). Of note, Cytosorb has been used in a pig model with combined severe smoke inhalation and burn trauma (40% body surface area) with promising first results (22). Additionally, the CytoSorb technology has meanwhile been approved for the removal of bilirubin as well as myoglobin, and latest data point toward beneficial effects in pathologies associated with increased levels of these substances (23, 24).

This technical note describes the principles of the first use of CytoSorb® therapy to promote the wound healing after severe burn trauma by reducing bilirubin levels and is based on a descriptive data collected in a single patient.

## Materials and Equipment

**Figure 1:**
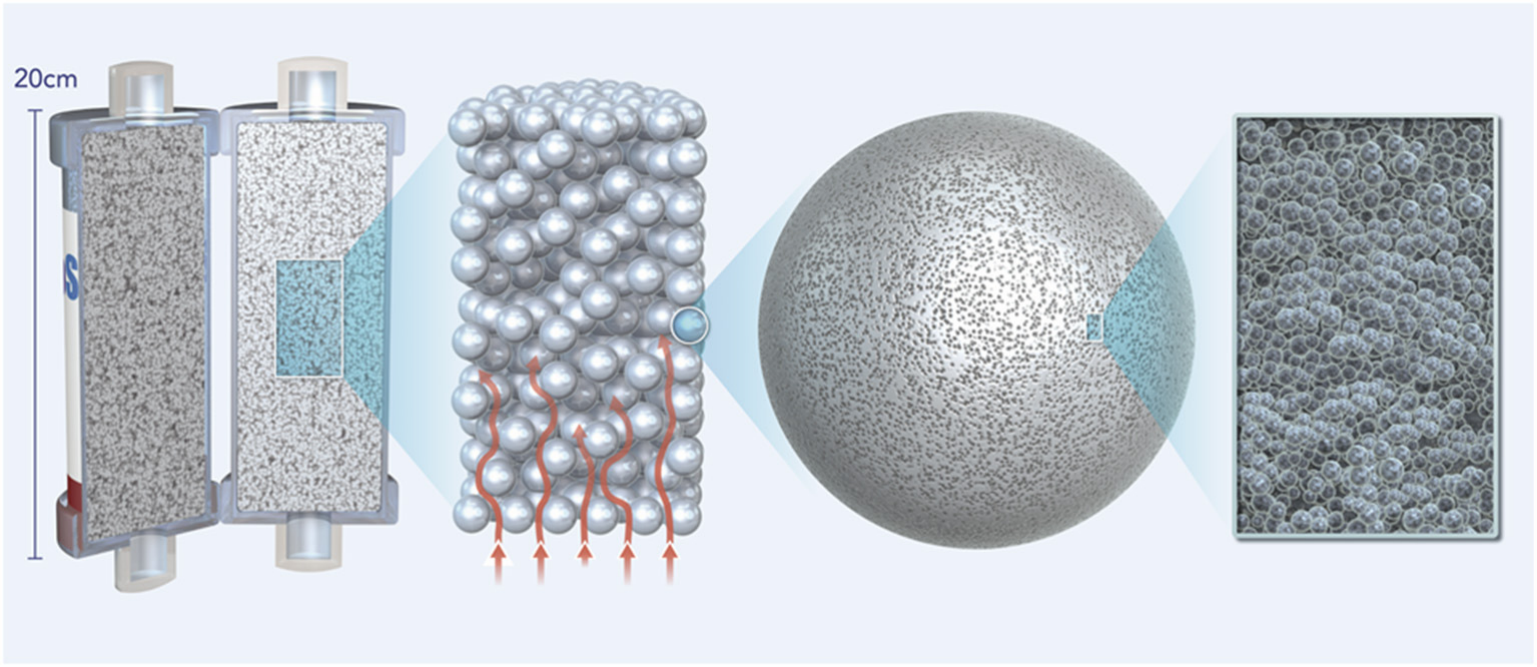
Figure showing the active matrix of the CytoSorb adsorber consisting of highly biocompatible porous polystyrene divinylbenzene (PSDVB) copolymer beads covered in a biocompatible polyvinylpyrrolidone coating.

A novel extracorporeal treatment, CytoSorb® (CytoSorbents Corp, USA), has been developed to remove excess molecules, which are involved in many clinical pictures and conditions of organ dysfunction, directly from blood. CytoSorb is a whole blood adsorber intended to be used as an adjunct to standard therapy in subjects where cytokines are elevated such as in the setting of hyperinflammation or septic shock. The active matrix consists of highly biocompatible and porous polystyrene divinylbenzene (PSDVB) copolymer beads approximately the size of a grain of salt covered in a biocompatible polyvinylpyrrolidone coating, with each bead containing millions of pores and channels capable of extracting mainly hydrophobic middle-sized (up to 55 kDa) molecules from blood *via* size exclusion (Figure 1). Binding is based in particular on hydrophobic non-covalent bonds. Blood purification by haemadsorption is broad-spectrum due to the non-selective and concentration-dependent removal of both pro- and anti-inflammatory mediators and is auto-regulating in that solutes at higher concentrations are removed more rapidly than those at lower, safer concentrations.

The adsorber can be easily integrated into an extracorporeal circuit for continuous renal replacement therapy (CRRT) which is important as renal failure frequently complicates liver failure. There is good evidence from the literature that the CytoSorb device is capable of removing compounds such as ammonia, bile acids and bilirubin from the blood compartment (25–29). Importantly, *in vitro* studies confirm superior elimination kinetics of the device compared to conventional liver support therapies such as the molecular adsorption recirculating system (MARS) (23, 30–32).

## Methods

**Figure 2:**
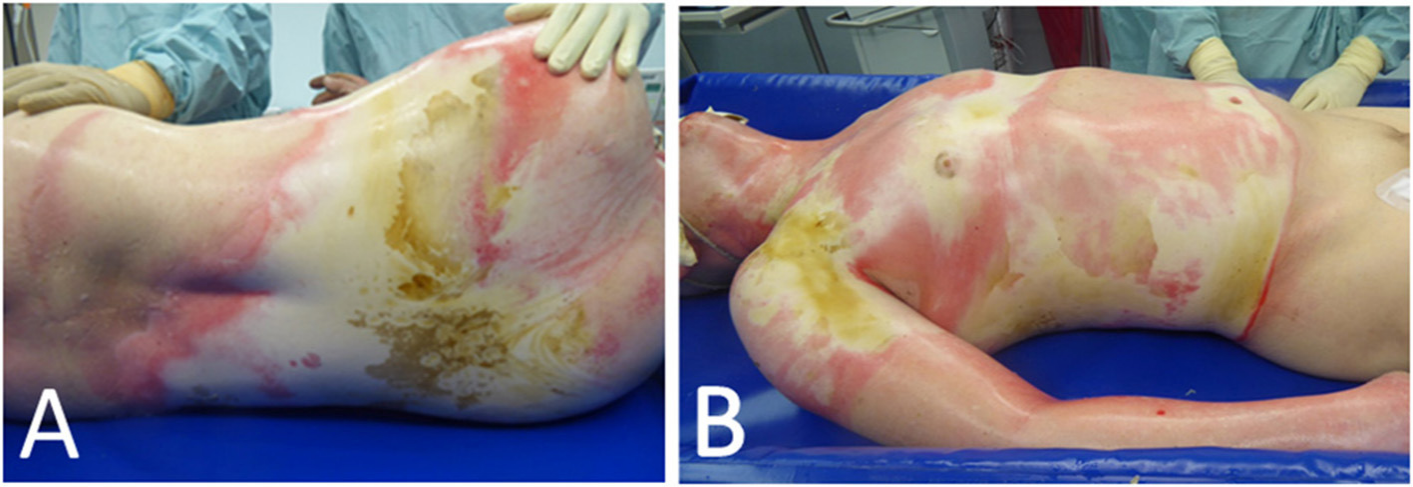
Extent of an initial burn trauma with 48% TBSA of the back **(A)** as well as upper extremities, abdomen and thorax **(B)** *via* electric arc.

We performed the therapy with CytoSorb on an initially healthy 59-year-old patient after a serious burn trauma involving head, upper extremities, thorax, upper abdomen and back with an extent of 48% TBSA (22% 2a-b°, 26% 3°) *via* electric arc, to promote healing of autologous skin grafts after multiple failed skin transplantations (Figure 2). Initially, we performed an escharotomy of the thorax as well as a tracheotomy on admission and, on the second day and 2.5 weeks after the trauma, epifascial debridement of the thorax, abdomen and extremities with autologous skin grafting.

**Figure 3:**
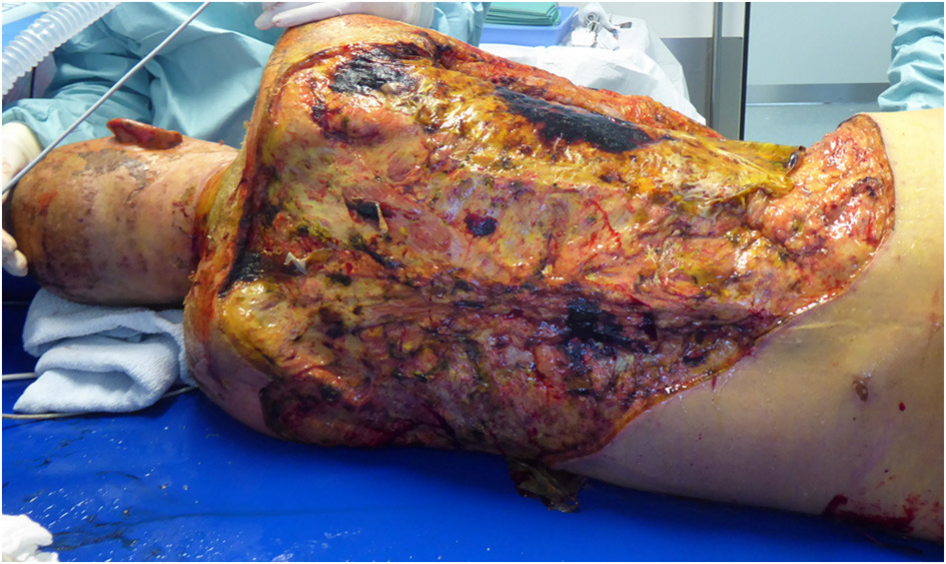
Serious wound complication of the back after removal of xenograft due to infection with MRSA and Candida albicans in an icteric patient.

Two weeks after the trauma, the patient developed HIT II (heparin-induced thrombocytopenia) and kidney failure, so that the CRRT was started. Anticoagulant therapy with heparin was converted to argatroban. Five days after the HIT diagnosis, HSV pneumonia and, 2 days later, Candida albicans and Proteus mirabilis sepsis were diagnosed by blood cultures. Early and aggressive treatment with wide spectrum antimicrobial, antimycotic and antiviral agents was performed. The cholestasis parameters (alkaline phosphatase-ALP, bilirubin), accompanied by aspartate transaminase (AST/GOT) had already started to rise steadily before the septic event and bilirubin reached 10.5 mg/dl 4 weeks after the initial trauma. Mechanical cholestasis and preexistent liver diseases were excluded. 3.5 weeks after admission, subfascial debridement of the back and xenograft transplantation were performed, as the previous autologous skin transplantation had failed, and the cholestasis parameters continued to trend upwards. Soon after, the patient developed a serious wound and xenograft infection with MRSA and Candida species (Figure 3); thus, the xenograft had to be removed, multiple daily wound debridements were performed and the antimicrobial as well as antifungal therapy was continued. Forty days after the injury, we performed MRCP (magnetic resonance cholangiopancreatography) that showed multiple biloma and intrahepatic cholestasis with a further increase in cholestasis parameters (50 days after the trauma bilirubin reached 17 mg /dl, conjugated bilirubin 14.9 mg/dl, ALP 275 U/l, and GGT 40 U/l). The hepatologists, however, did not rule out liver regeneration or that the patient could eventually be a candidate for liver transplantation in the future. Therefore, we aimed to reduce the massive wounds in a timely fashion with the help of CytoSorb therapy so that the bilirubin levels decreased, as in our daily practice, the failure of skin grafting was frequently observed in icteric patients. Our rationale for using CytoSorb therapy was, in particular, based on experimental studies which showed a negative influence of bilirubin on fibroblast proliferation and collagen formation (1–5). It was also based on observations of previous authors that icteric patients after abdominal surgery and severely burned patients with hyperbilirubinaemia were at risk of wound healing impairment (6–8).

## Results

**Figure 4:**
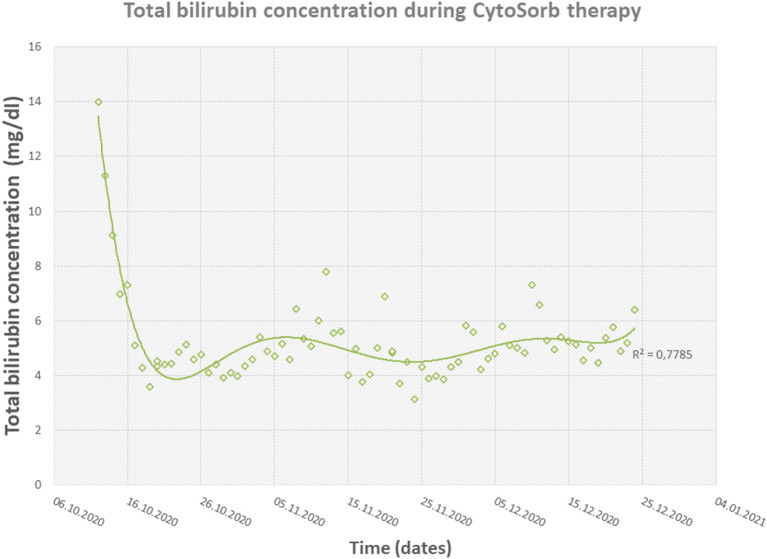
Diagram showing the fast reduction in total bilirubin concentrations during CytoSorb therapy.

**Figure 5:**
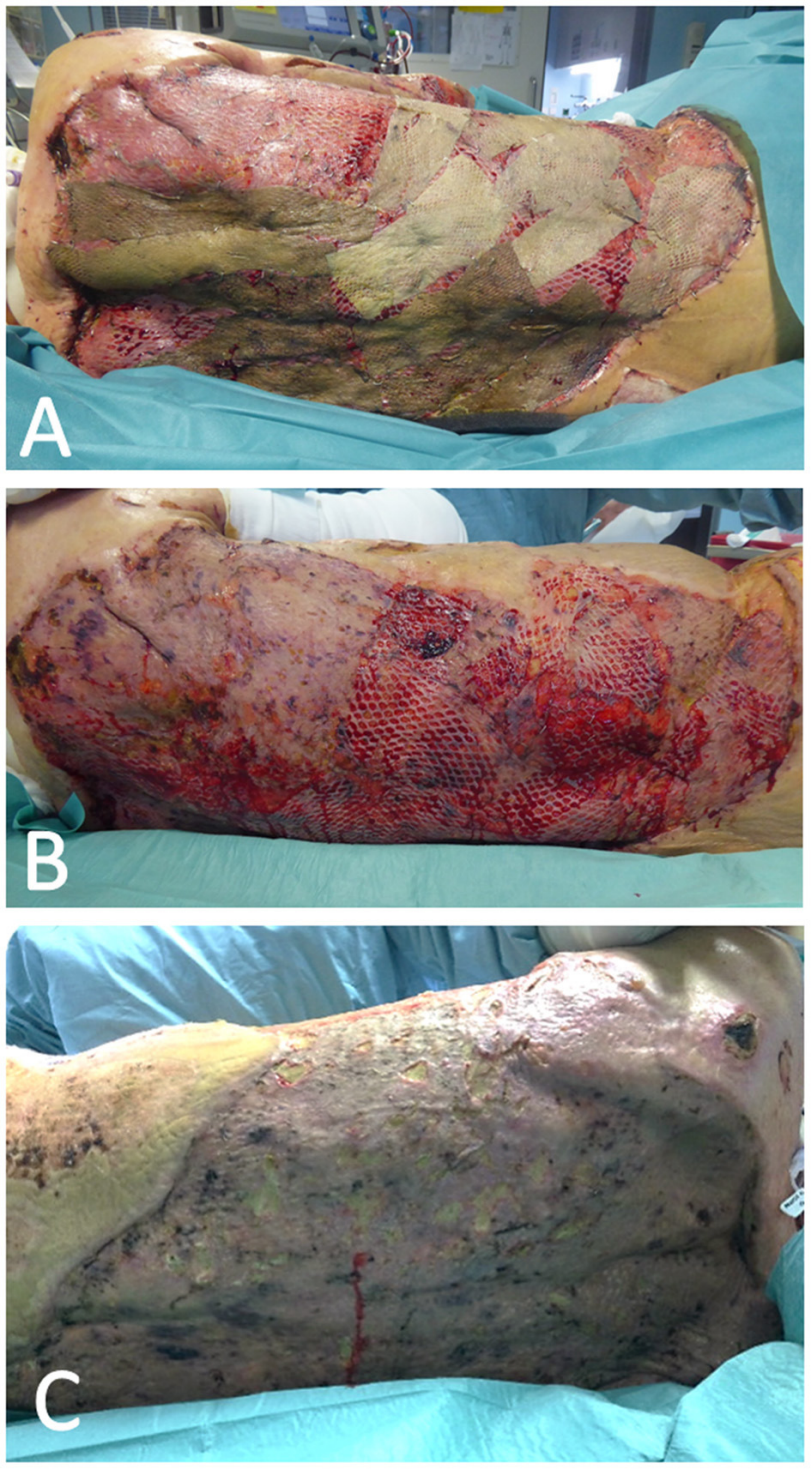
Satisfying result of debridement and autologous skin grafting after a decrease in bilirubin concentration following the use of CytoSorb. An allogenous skin graft was used as a protective layer **(A)** and was removed about 10 days after surgery, leaving only an autologous graft **(B)**, which showed good integration and wound closure with only small defects 4 weeks after the surgery **(C)**.

The therapy with CytoSorb was initiated on day 52 after admission with a total bilirubin count of around 14 mg/dl. In the first 6 days, the cartridge was changed on a daily basis and later after every 2–4 days. The therapy with six adsorbers after 6 days reduced the total bilirubin level from 14.02 to 4.29 mg/dl (Figure 4). With a stable bilirubin level under 5 mg/dl 1 week after the CytoSorb therapy began, debridement of abdomen and upper extremities with autologous skin grafting was performed successfully. Four weeks later, under CytoSorb therapy, debridement of the back with autologous skin grafting from scrotum and lower extremities with allogenous skin grafting as a protective layer was performed under supplementation with coagulation factors and thrombocytes (Figure 5A). The intraoperative substitution of red blood cell concentrates transiently increased the total bilirubin level to 6.89 mg/dl, which was again reduced with a CytoSorb cartridge change on the day of the operation and on the following day and later after every third day. This allowed a bilirubin reduction to less than 5 mg/dl on the first day postoperatively.

After the removal of the allogenous protective skin layer the autologous skin grafts showed a near-total take (Figures 5B,C). Eight days after the last operation, as the catecholamine demand rose, blood cultures were sampled and sepsis with Candida parapsilosis was diagnosed, even though the antifungal therapy with anigulafungin was continued according to the antibiogram results. In a CT-directed biopsy of the bilomas, intrahepatic cholestasis with intrahepatic cholangitis and bilous interposed necrosis as well as infection with Candida albicans was diagnosed. Based on the histopathological findings and persistent cholestasis the hepatologist confirmed the SCC diagnosis. After wound healing had been reached, CytoSorb therapy after 57 days and 27 adsorber changes was discontinued. Four weeks later bilirubin level rose again to 8.2 mg/dl but did not reach the maximal peak seen before the CytoSorb therapy began.

## Discussion

We herein report the case of a severely burned (48% TBSA) patient with SCC and hyperinflammation in which we applied CytoSorb haemoadsorption to promote the wound healing process by controlling the generalized inflammatory response and by reducing bilirubin concentrations in order to bridge the time to spontaneous liver regeneration or eventually to liver transplantation after two skin transplantations had failed to provide wound closure. Treatment resulted in a decrease in total bilirubin plasma concentrations enabling successful surgical therapy of the abdomen, back as well as lower and upper extremities within 4 weeks following CytoSorb treatment.

Our report therefore represents one of the first descriptions of the application of the CytoSorb haemoadsorption column in severe burn trauma with apparently promising results. Houschyar et al. described the case of a 21-year-old patient admitted to the hospital immediately following an explosion at home with 2b-3-degree burns to a total of 60% of the TBSA (33). Multiple operations were performed, while further therapy consisted of epifascial debridements, keratinocyte deposits and automatic prone/supine positioning. Due to sustained elevation of the inflammatory parameters (leukocytes, CRP and PCT), positive blood cultures and wound smears for Acinetobacter baumannii, the decision was made to start hemofiltration therapy with additional CytoSorb adsorbers resulting in control of the hyperinflammatory response and haemodynamic stabilization.

In the case of SCC, therapeutic options are limited to liver transplantations (LT) while patients not undergoing transplantation show significantly reduced survival compared with patients with primary sclerosing cholangitis (idiopathic form mostly associated with inflammatory bowel disease). Sclerosing cholangitis in critically ill patients is, therefore, associated with rapid disease progression and poor outcome with estimated mortality rates after 6 months being higher than 50% (15, 34). In the case of severely burned patients, liver transplantation is frequently not possible because of open wounds and ongoing infections. Therefore, timely wound closure is of great importance.

Although there is much anecdotal evidence to suggest that jaundice impairs wound healing, experimental studies have failed to definitely pinpoint its causes, as the models of obstructive jaundice cause not only hyperbilirubinaemia but also hypoproteinaemia, anemia and dysfunction of liver synthesis. Bilirubin was, however, shown to be a metabolic poison in studies on purified respiratory enzymes and tissue culture systems. Bilirubin inhibits the NADH2 oxidase, uncouples the oxidative phosphorylation, and decreases ATP production, cell viability as well as amino acid incorporation in proteins (35, 36). Taube et al. showed that the addition of unconjugated bilirubin to the culture medium with fibroblasts caused morphological changes in the cells and significantly decreased their rate of multiplication (4, 5). Arnaud et al. showed in a rat model that abdominal wall resistance in icteric animals that fibroblast migration was reduced (1). On the other hand, in the latest experimental studies, bilirubin at lower concentrations applied topically seemed to facilitate wound healing in diabetic rats by modulating growth factors and promoting angiogenesis as well as collagen formation (37).

Wound complications after abdominal surgery have been shown to be generally more common in icteric cases than in anicteric patients. Also, a significantly lower activity of skin prolylhydroxylase as an index of collagen formation was found to be related to the high incidence of wound dehiscence in icteric patients (7, 8). Liu et al. reported a strong clinical association between high bilirubin levels and impaired wound healing in severely burned patients (6). We did not find any example in the literature on the use of the CytoSorb adsorber to improve wound or skin graft healing in cases of hyperbilirubinaemia. Many authors, however, have used the CytoSorb therapy for biochemical improvement in the case of liver failure (25–29).

Gemelli et al. observed a removal ratio of bilirubin of up to 56% over 8 h in an extracorporeal *in vitro* circuit with CytoSorb (31). The molecules of CytoSorb, due to their strong hydrophobicity, are able to break the strong albumin-bilirubin complex and irreversibly adsorb bilirubin, leaving albumin in the solution (22). Calabro et al. showed in a study on 40 critically ill patients a significant reduction in bilirubin concentration with 2–4 CytoSorb adsorbers in cases of an initial hyperbilirubinaemia of at least 10 mg/dl (38). Tomescu et al. observed a decrease in bilirubin and ammonia levels and decrease of CRP after three sessions of continuous venovenous haemofiltration in combination with CytoSorb (39). Although a subsequent decrease of platelet count was observed, it did not result in any haemorrhagic complications.

A latest case series reports on the use of CytoSorb therapy in 33 patients with liver dysfunction from Scharf and coworkers reports on a significant bilirubin reduction during CytoSorb treatment accompanied by significant decreases of AST, alanine aminotransferase and gamma-glutamyl transferase, reflecting an accompanying improvement in the overall liver function, as direct adsorption is unlikely due to molecular size of these substances (>64 kDa) (23). Furthermore, treatment was associated with a lower-than-expected mortality (92 vs. 82.2%), a significant reduction in norepinephrine requirements resulting in haemodynamic stabilization as well as a significant reduction in SAPS II (Simplified Acute Physiology Score) during haemoadsorption treatment as another indicator of an improvement in the patient's outcome.

Last but not least, also ongoing hyperinflammatory processes can have a negative impact on wound healing (40). In an interim analysis of a prospective randomized controlled trial on the intraoperative use of CytoSorb therapy in cardiac surgery to assess the effects of preventive immunomodulation, the rate of sternal wound healing disorders appeared to be reduced in the CytoSorb treated patients (41). To which extent addressing hyperinflammation might have also contributed to the present case remains of course purely speculative.

Although wound healing in our patient was reached, 1 month after therapy with CytoSorb had been discontinued, the patient died of severe pneumonia and systemic mycosis with Candida spp., which was sampled from the tracheal secretions, ascites, liver and urine catheter, and which did not respond to the antifungal therapy. Xiao et al. showed that nosocomial infections and organ failure in patients after severe burns and trauma could be associated with prolonged genomic changes, called a genomic storm, which activates simultaneously various pro-inflammatory and immunosuppressive pathways (42). Because the difference in gene expressions between complicated and uncomplicated courses was not qualitative but quantitative, the therapeutic approach reducing the amount of circulating cytokines and toxins could be used earlier in the case of burn trauma to decrease a non-resolving inflammation and improve patient outcome. Therefore, this approach might warrant further research in patients suffering from severe burns.

Our current study represents a single and case description with a technical note. Additionally, the lack of a control arm, and the use of various interventions (CytoSorb, fluids, blood transfusions, antimicrobial, and antimycotic agents) in the treatment of the patient account to major limitations of the report. Based on a single report, potential conclusions in terms of dosing and timing cannot be stated. Moreover, long-time observation of the patient was not possible, because of the lethal infectious complications. All of these limitations should be considered in the design of upcoming trials on a larger group of patients.

CytoSorb therapy showed improvement in the clinical results of wound healing in a patient with liver failure due to reduction of hyperbilirubinemia. Since therapy options for severely burned patients with impairment of wound healing due to serious liver dysfunction are very limited, the CytoSorb haemadsorption therapy can be a promising support for wound and skin graft healing in selected patients due to significant reductions in total bilirubin concentrations.

## Data Availability Statement

The raw data supporting the conclusions of this article will be made available by the authors, without undue reservation.

## Ethics Statement

Written informed consent was obtained from the individual(s) for the publication of any potentially identifiable images or data included in this article.

## Author Contributions

KR: conception or design of the work, data collection, data analysis and interpretation, and drafting the article. MK and JT: data collection, data analysis and interpretation. JK and AD: critical revision of the article and final approval of the version to be published. AB: conception or design of the work, critical revision of the article, and final approval of the version to be published. All authors contributed to the article and approved the submitted version.

## Funding

This work was funded by the company CytoSorbents.

## Conflict of Interest

The open access was funded by the company CytoSorbents. The authors declare that the research was conducted in the absence of any commercial or financial relationships that could be construed as a potential conflict of interest.

## Publisher's Note

All claims expressed in this article are solely those of the authors and do not necessarily represent those of their affiliated organizations, or those of the publisher, the editors and the reviewers. Any product that may be evaluated in this article, or claim that may be made by its manufacturer, is not guaranteed or endorsed by the publisher.
